# Rift Valley fever among febrile patients at New Halfa hospital, eastern Sudan

**DOI:** 10.1186/1743-422X-7-97

**Published:** 2010-05-13

**Authors:** Ahmed M Hassanain, Waleed Noureldien, Mubarak S Karsany, El najeeb S Saeed, Imadeldin E Aradaib, Ishag Adam

**Affiliations:** 1 Faculty of Medicine, University of Khartoum, Khartoum, Sudan; 2 New Half Teaching Hospital, New Halfa, Sudan; 3 Faculty of Medicine, Juba University, Khartoum, Sudan; 4 Molecular Biology Laboratory, Faculty of Veterinary Medicine, University of Khartoum, Khartoum, Sudan

## Abstract

**Background:**

Since the first isolation of the Rift Valley Fever virus (RVFV) in 1930s, there have been several epizootics outbreaks in the tropic mainly in Africa including Sudan. Recognition of cases and diagnosis of RVF are critical for management and control of the disease.

**Aims:**

To investigate the seroprevalence and risk factors for seropostive to RVFV IgG among febrile patients.

**Methods:**

All febrile patients presented to New Halfa hospital in eastern Sudan during September through November 2007 were investigated to identify the cause of their fever including malaria and RFV.

**Results:**

Out of 290 feverish patients presented to the hospital, malaria was diagnosis in 94 individuals. Fevers of unknown origin were diagnosed in 149 patients. Seropostive to RVFV IgG was detected by enzyme-linked immunosorbent assay in 122 (81.8%) of the sera from these 149 patients with fever of unknown origin. While socio-demographic characteristics (age, Job, education and residency) were not associated with seropostive to RVFV IgG, male (OR = 2.8, 95% CI = 1.0-7.6; *P *= 0.04) were at three times higher risk for seropostive to RVFV IgG.

**Conclusion:**

There was a high seropostive to RVFV IgG in this setting, more research is needed perhaps using other methods like PCR and IGM.

## Introduction

The Rift Valley Fever virus (RVFV) of the family *Bunyaviridae *is a cause of zoonotic viral disease [1]. Since the first isolation of the virus in1930s, there have been several epizootics outbreaks in tropic mainly in Africa including Sudan, which is the largest country in Africa [2,3]. RVFV Infection in humans can be acquired through mosquito bites, through contact with infected animals and vertical transmission has been reported [4]. RVF can present as uncomplicated acute febrile illness, however severe complications, such as hemorrhagic disease, meningoencephalitis, renal failure and blindness have been reported [2,5,6]. Generally, it has been estimated that only approximately 1%-2% of infections result in fatal hemorrhagic fever [7]. It has been reported that significant high-prevalence clusters of RVF encompassed areas that had experienced previous epidemics of RVF [8].

RVF and other arthropod-borne pathogens as the cause of an outbreak of febrile illnesses were reported previously, following previous flooding in the different regions of Sudan [9-11]. Furthermore, recently RVF causing outbreak in has been reported in Sudan [2,3]. The importance of recognition of cases and diagnosis, especially in malaria endemic areas, of these viruses are critical for management and control of the disease. Hence, effective countrywide surveillance backed by diagnosis is highly recommended. Due to the on-going climatic changes, such epidemic-outbreaks are expected to occur following the rainy season. According to our experience in New Halfa area, febrile illness and malaria are the major health problems [12,13]. It is worth mentioning that not all of these are malaria cases, hence it would be of paramount importance to conduct surveys for RVF [12,13]. Strengthened surveillance, early detection, management of cases seemed to be among the best options to prevent extension of RVF epidemic foci. Precise estimation of specific weight for each risk factor is a considerable guide to construct an effective outbreak control plan. Thus the objective of the present study was to investigate the prevalence and risk factor -if any- for RVF among febrile patients presented at New Halfa Hospital in eastern Sudan.

## Methods

The study was conducted in New Halfa hospital in eastern Sudan during October through December 2007 to investigate the seroprevalence and risk factors for RVFV among febrile patients. The hospital served around 500000 populations in New Hlafa, eastern Sudan. This area is located at 500 km from Khartoum in the middle of the second largest irrigated agricultural scheme in Sudan. Cotton and wheat are the main crops cultivated during the winter season. The region is semi arid dry of Savannah belt of Sudan characterized by mean temperature of 29.4°C (range 14.1-42.7°C). After signing an informed consent, detailed medical history was gathered by the physician from all febrile patients (temperature ≥ 37.5°C) using questionnaires. Then medical history and physical examinations including the vital sings were followed by suitable optimum investigations e.g. chest x-ray, urine analyses, urine culture and sensitivity, Widal test for typhoid, paratyphoid and brucellosis and blood film for malaria.

A suspected human RVF case-patient was defined as a person with fever associated or not with hemorrhagic jaundice, and neurological symptoms. A confirmed human RVFV case-patient was defined as immunoglobulin G (IgG). For each case, blood samples were collected and an interview in which information was gathered about sex, age, date of fever onset, profession and hemorrhagic symptoms-if any- for all patients.

## Ethics

The study received ethical clearance from the Research Board at the Faculty of Medicine, University of Khartoum, Sudan.

## Statistics

The data were entered in computer using SPSS for window (version 13.0) and double checked before analyses. Frequencies were calculated. Logistic regression analyses were performed using the seropostive to RVFV IgG as dependent variable and the socio-demographic characteristics as independent variables. Odd ratios and 95% confidence interval were calculated and *P *< 0.05 was considered significant.

## Results

Out of 290 patients with fever presented to the hospital, diagnosis of malaria, based primarily on clinical presentation was made in 94 individuals. Thirty two and 24 patients had respiratory and urinary tract infections, respectively. Fevers of unknown origin were diagnosed in 149 patients and some patients had mixed infections. Seropostive to RVFV IgG was detected by enzyme-linked immunosorbent assay in 122 (81.8%) of the sera from these 149 patients with fever of unknown origin.

Different symptoms were observed among these 149 patients e.g. fever, sweating, headache, chills. None of the patients presented with hemorrhagic symptoms and there was no death. Out of these149 patients, 107 (71.8%) were male, 60(40.3%) were illiterate, 80(53.7%) were rural residence. The mean (SD) of these 149 patients was 36.6(13.8) years and the mean (SD) of their illness was 6.1 (4.5) days.

### Factors associated with seropostive to RVFV IgG

While socio-demographic characteristics (age, Job, education and residency) were not associated with seropostive to RVFV IgG, male (OR = 2.8, 95% CI = 1.0-7.6; *P *= 0.04 were at three times higher risk for seropostive to RVFV IgG, table 1.

**Table 1:** Showing logistic regression analysis for seropostive to RVFV IgG in New Halfa hospital, eastern Sudan.

The variables	OR	95% CI	*P*
Age	1.0	0.9-1.0	0.2
Gender, male	2.8	1.0-7.6	0.04
Job	1.9	0.7-5.6	0.19
Residency	1.9	0.7-5.4	0.1
Education	2.1	0.7-5.9	0.1

## Discussion

The main findings of the current study were; the high prevalence of seropostive to RVFV IgG in the area and male were at three times higher risk for RVF. RVF outbreaks usually occur during the seasons of high rainfall when the mosquito population is abundant. The periods between the outbreaks may extend to several decades during which it is difficult to diagnose cases of RVFV infection except with special epidemiologic and laboratory techniques. Antibodies to RVFV infection can be diagnosed by detection of IgG antibodies to RVFV in the serum. Thus, suspect cases can be observed through active surveillance and diagnosis can be confirmed by detection of IgM antibodies. Although virus isolation is considered as gold standard method, IgM-ELISA method avoids false positive results due to the presence of rheumatoid factor and antinuclear antibodies. On the other hand, anti-RVFV antibodies were estimated to persist at a detectable level for long time in chronic infections [14]. Thus, combination of ELISA and PCR assays is very important for rapid and efficient identification of RVFV during outbreaks. The data obtained during the epidemics of RVF in neighboring in Kenya [15], as well as in Saudi Arabia and Yemen [16] demonstrated the importance of combining diagnostic assays for accurate and comprehensive detection of RVFV infection.

In the current study there were various symptoms and there were no hemorrhagic manifestations among these patients. Recently we observed various severe manifestations of RVF in the central Sudan [2] and Seufi and Galal observed RVFV in the different region of Sudan among the mosquitoes and human being as well [17].

In the current study age and job were not predictors for seropostive to RVFV IgG. However, males were observed to have higher risk for severe RVF in central Sudan [2] as well as in this study. Previous results from different region of Sudan indicated that males of 15-29 years old were more susceptible to RVF than females. In parallel, housewives and farmers were the most susceptible people to RVF infection. These results may be related to their more vulnerability to the vector as well as to socioeconomic/professional activities which allow a direct contact with infected animals. Woods et al observed that children < 15 years of age were less likely to have had RVFV infection [15]. One of the limitations of the study was that, IgG was diagnostic tool for RVFV. IgG is the only indicator of the person exposure to RVFV and may not be suitable candidate test for detection recent infections. Capture IGM is the suitable one. Because of fund constrains we did not performed IGM as we did before in our previous reports [2]. Actually this study was conducted to see if RVFV was involved as cause of febrile illnesses in eastern Sudan as well as other parts or not?. Thus strengthened surveillance, early detection, management of cases seemed to be among the best options to prevent extension of RVF epidemic foci. Precise estimation of specific weight for each risk factor is a considerable guide to construct an effective outbreak control plan.

## Conclusion

There was high prevalence of seropostive to RVFV IgG in this setting, more research is needed perhaps using other methods like PCR and IGM.

## Competing interests

The authors declare that they have no competing interests.

## Authors' contributions

AMH and IA designed the study. WN and AMH conducted the clinical work. MSK, NS and IEA performed the laboratory work. IEA and IA analyzed the data. All the authors shared in the drafting of the paper and all of them approved the paper.
